# Trypanosomiasis-Induced Th17-Like Immune Responses in Carp

**DOI:** 10.1371/journal.pone.0013012

**Published:** 2010-09-27

**Authors:** Carla M. S. Ribeiro, Maria J. S. L. Pontes, Steve Bird, Magdalena Chadzinska, Marleen Scheer, B. M. Lidy Verburg-van Kemenade, Huub F. J. Savelkoul, Geert F. Wiegertjes

**Affiliations:** 1 Cell Biology and Immunology Group, Department of Animal Sciences, Wageningen University, Wageningen, The Netherlands; 2 School of Biological Sciences, Scottish Fish Immunology Research Centre, University of Aberdeen, Aberdeen, United Kingdom; 3 Department of Evolutionary Immunobiology, Institute of Zoology, Jagiellonian University, Kraków, Poland; Duke University Medical Center, United States of America

## Abstract

**Background:**

In mammalian vertebrates, the cytokine interleukin (IL)-12 consists of a heterodimer between p35 and p40 subunits whereas interleukin-23 is formed by a heterodimer between p19 and p40 subunits. During an immune response, the balance between IL-12 and IL-23 can depend on the nature of the pathogen associated molecular pattern (PAMP) recognized by, for example TLR2, leading to a preferential production of IL-23. IL-23 production promotes a Th17-mediated immune response characterized by the production of IL-17A/F and several chemokines, important for neutrophil recruitment and activation. For the cold blooded vertebrate common carp, only the IL-12 subunits have been described so far.

**Methodology/Principal Findings:**

Common carp is the natural host of two protozoan parasites: *Trypanoplasma borreli* and *Trypanosoma carassii*. We found that these parasites negatively affect p35 and p40a gene expression in carp. Transfection studies of HEK293 and carp macrophages show that *T. carassii*-derived PAMPs are agonists of carp TLR2, promoting p19 and p40c gene expression. The two protozoan parasites induce different immune responses as assessed by gene expression and histological studies. During *T. carassii* infections, in particular, we observed a propensity to induce p19 and p40c gene expression, suggestive of the formation of IL-23. Infections with *T. borreli* and *T. carassii* lead to an increase of IFN-γ2 gene expression whereas IL-17A/F2 gene expression was only observed during *T. carasssii* infections. The moderate increase in the number of splenic macrophages during *T. borreli* infection contrasts the marked increase in the number of splenic neutrophilic granulocytes during *T. carassii* infection, along with an increased gene expression of metalloproteinase-9 and chemokines.

**Conclusion/Significance:**

This is the first study that provides evidence for a Th17-like immune response in fish in response to infection with a protozoan parasite.

## Introduction

In mammalian vertebrates, the three members of the IL-12 cytokine family comprise the heterodimeric IL-12, IL-23 and IL-27; all molecules belonging to the type I cytokine superfamily. IL-12 consists of two disulphide-linked proteins: the p35 and the p40 subunit, together forming IL-12p70 [1]. The p40 subunit also participates in the formation of IL-23, formed in combination with a subunit related to p35, named p19 [2]. The last member of the family, IL-27, consists of a heterodimer between two other subunits, although both with homology to the p35 and p40 subunits, respectively, named p28 and EBI3 [3]. Because IL-12 and IL-23 share the IL-12Rβ1 receptor subunit, the ability of cells to respond to either IL-12 or IL-23 is determined by the expression of the other chain; IL-12Rβ2 or IL-23R, respectively [4], [5]. IL-27 signals also through a heterodimeric receptor composed of IL-27Rα and gp130, the latter is shared with the IL-6 receptor [3]. Thus, despite their structural similarities, based on recognition by distinct heterodimeric receptors on the cell membrane, IL-12, IL-23 and IL-27 have distinct functions.

For the cold blooded vertebrate common carp (*Cyprinus carpio* L.), not all IL-12 cytokine family members have been described. Sequences for both p35 and p40 were found, suggestive of the formation of an IL-12 molecule in carp. The subunit p35 so far was reported as a single gene, whereas three distinct p40 genes (p40a, p40b and p40c) were described [6], [7]. Major differences between the three p40 isoforms in constitituve gene expression and *in vitro* inducibility, indicate differences in function. Of the three carp p40 isoforms, p40a seems most similar to mammalian p40 proteins with respect to amino acid sequence identity and conservation of critical residues for heterodimerisation with p35 [6]. Upregulation of gene expression of p35 and p40b, in particular, is suggestive of the formation of an IL-12-like molecule as a signal-3 cytokine during viral infection [8]. With regard to the third isoform, p40c, phylogenetic analysis indicates that p40a and p40b share a common ancestor following their divergence from p40c. More importantly, the cysteine residue in the human p40 molecule that forms a stabilizing interchain disulphide bridge with p35 is not present in carp p40c [6]. Only very recently the subunit p19 was described as a molecule likely involved in the IL-23/Th17-driven immune response in zebrafish (*Danio rerio*) [9]. Carp and zebrafish are closely-related species that belong to the same Family (Cyprinidae) and have similar immunological responses. In the present manuscript, we studied gene expression of different IL-12 cytokine family members, including p19, p35 and p40a–c, in response to protozoan infection of carp.

In mammalian vertebrates, IL-12 constitutes an important factor in the differentiation and expansion of Th1 cells, which produce IFN-γ that activates myeloid cells to secrete TNF-α, both essential for defense against intracellular pathogens. IL-23 is required for the expansion of Th17 cells, which produce IFN-γ but also IL-17A and IL-17F, essential for the defense against extracellular bacteria and fungi [10], [11]. Thus, Th17-mediated immune responses are IL-23 driven and characterized by the production of IL-17A/F and several chemokines, all important for neutrophil recruitment and activation [12]. During infections, the balance between IFN-γ and IL-17A/F cytokines represents the balance between a Th1 and a Th17 response [13]. In carp, IFN-γ exists as two distinct genes with IFN-γ2, in particular, being associated with characteristic T-lymphocyte function and classical phagocyte activation. The exact function of IFN-γrel (previously referred to as IFN-γ1) is presently unknown. Zebrafish IL-17A/F exists as three distinct genes with IL-17A/F2, in particular, being the only IL-17A/F gene expressed in both systemic (kidney) and mucosal (intestine and gills) immune tissue [14]. In the present manuscript, we describe the possibility that formation of carp IL-23 can lead to a Th17-like immune response to a protozoan parasite of common carp.

Professional antigen presenting cells are the prime source of all three IL-12 cytokine family members, at least in mammalian vertebrates. The balance between the three IL-12 cytokine family members during the course of an immune response is dependent on the nature of the pathogen associated molecular pattern (PAMP) recognized [15], [16], [17]. For example in mammalian vertebrates, TLR2 activation by bacterial-derived peptidoglycan (PGN) induces high levels of IL-23 but not IL-12 [15]. We recently identified carp TLR2 and described its involvement in the recognition of bacterial PAMPs such as PGN but also of GPI-anchors from protozoan parasites [18]. Carp is the natural host of two extracellular protozoan blood parasites: *Trypanoplasma borreli* and *Trypanosoma carassii*. Both *T. borreli* and *T. carassii* are believed to live extracellular in the blood and tissue fluids of their fish hosts [19] [20]. The immune response of carp against these two parasites is fundamentally different. *T. borreli* infections are associated with a type 1-like immune response, characterized by a classical IFN-γ2-mediated activation of macrophages leading to a high production of TNF-α and NO within 3 weeks [21], [22], [23]. In contrast, *T. carassii* infections of carp do not lead to an excessive NO response, extend over a 2–3 weeks longer infection period and are associated with an alternative activation of macrophages [22], [24], [25]. In the present manuscript, we describe that *T. carassii*, in particular, induces a Th17-like immune response in carp.

## Results

### Live protozoan parasites induce TLR2 gene expression in carp macrophages

Glycosylphosphatidylinositol (GPI) anchors (or their fragments) from protozoan parasites have been shown to trigger TLR2 activation [26], [27]. We made use of the fact that bacterial phosphatidylinositol-specific phospholipase C (PI-PLC) can cleave GPI-anchors in eukaryotic cells. The cleavage promotes the release of soluble proteins into a supernatant now containing free GPI-phospholipids and diacylglycerol. The supernatant was used to study the possibility that GPI-anchored proteins from *T. borreli* and *T. carassii* can act as PAMPs of carp TLR2. Carp macrophages, when stimulated with supernatant from PI-PLC-treated *T. borreli* protozoan parasites as a source of GPI-anchors, show a 2–3 fold up-regulation of TLR2 gene expression [18]. GPI-anchors from another protozoan parasite of carp, *T. carassii*, also induced a dose-dependent up-regulation of TLR2 gene expression (Table 1). Negative controls including non-PI-PLC-treated parasites and pellets from PI-PLC-treated parasites did not significantly modulate gene expression of TLR2 (data not shown). *In vitro* stimulation with live *T. borreli*, and to a lesser extent live *T. carassii*, resulted in an approximate 2 fold up-regulation of TLR2 gene expression in carp macrophages.

**Table 1:** TLR2 fold change in carp macrophages after 6 h stimulation with live or PI-PLC-treated parasites.

	*T. borreli* a	*T. carassii* a
**PI-PLC-treated (millions)**		
1	1.73	1.21
2.5	1.63	1.28
5	2.91*	1.44*
**Live (millions)**		
0.1	1.26	0.90
0.25	1.50	1.26
0.5	1.92	1.55

a Averages of four fish are shown.

*Significant (*P*≤0.05) difference in gene expression compared to unstimulated cells.

mRNA levels of TLR2 were determined by real-time quantitative PCR analysis. mRNA levels of TLR2 were normalized against the house keeping gene 40S ribosomal protein S11 and are shown as fold change relative to unstimulated macrophages.

### Protozoan-derived PAMPs are ligands of carp TLR2

HEK 293 cells were used to investigate protozoan parasite ligands for carp TLR2 using MAPK-p38 phosphorylation as a measure for responsiveness. HEK 293 cells were transfected with carp TLR2 full length (TLR2 WT) or with carp TIR-domain truncated TLR2 (TLR2ΔTIR). MAPK-p38 phosphorylation in transfected HEK 293 cells was detected using an antibody specific for phospho-p38 [18].

**Figure 1:**
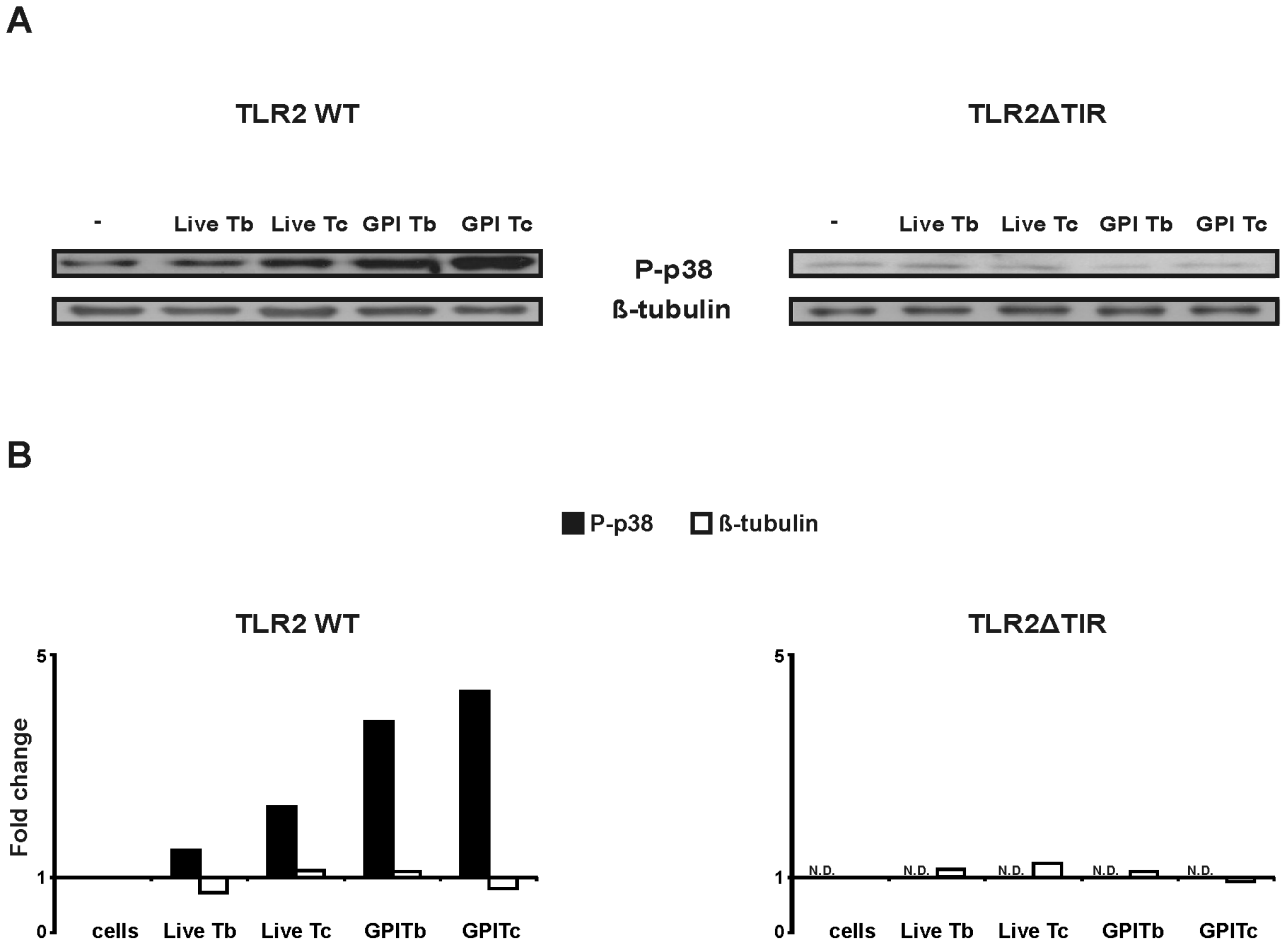
Activation of carp TLR2 by parasite-derived PAMPs in HEK 293 cells. TLR2WT- and TLR2ΔTIR-transfected HEK 293 cells were stimulated with live *T. borreli* or *T. carassii* parasites (Live Tb or Live Tc, 0.5 million parasites per well), with supernatants from 5 million PI-PLC-treated- *T. borreli* or -*T. carassii* parasites (GPI Tb or GPI Tc) or left untreated as negative control. MAPK-p38 phosphorylation was analysed by immunoblotting for phospho-p38 (P-p38), while equal loading was confirmed by immunoblotting for β-tubulin (**A**). Fold change of P-p38 and β-tubulin protein levels in the stimulated cells of TLR2WT- and TLR2ΔTIR-transfected HEK 293 cells are shown relative to the untreated cells. The P-p38 signal intensity of TLR2ΔTIR-transfected HEK 293 cells was below the threshold and therefore non-detectable (N.D.) (**B**). One experiment representative of three independent experiments is shown.

Stimulation of HEK 293 cells transfected with truncated TLR2 (TLR2ΔTIR) never increased MAPK-p38 phosphorylation (TLR2ΔTIR, Fig. 1) when stimulated with GPI-anchors from protozoan parasites (supernatant from PI-PLC-treated) nor when stimulated with live parasites. This negative control thereby showed the unresponsiveness of the parental HEK 293 cells to the protozoan parasite derived PAMPs. In contrast, stimulation of TLR2 WT-transfected HEK 293 cells increased MAPK-p38 phosphorylation, both with a source of GPI-anchors from both parasites and with live (*T. carassii*) parasites as stimulants (TLR2 WT, Fig. 1A, B). The magnitude of the response was different between the two protozoan parasites with GPI-anchors from *T. carassii* inducing a higher MAPK-p38 activation than GPI-anchors from *T. borreli*. Thus, transfection of HEK 293 cells with TLR2 WT established the ability of carp TLR2 to recognize protozoan parasite, in particular *T. carassii*-derived PAMPs.

### Protozoan parasites induce or suppress different members of the IL-12 cytokine family

**Figure 2:**
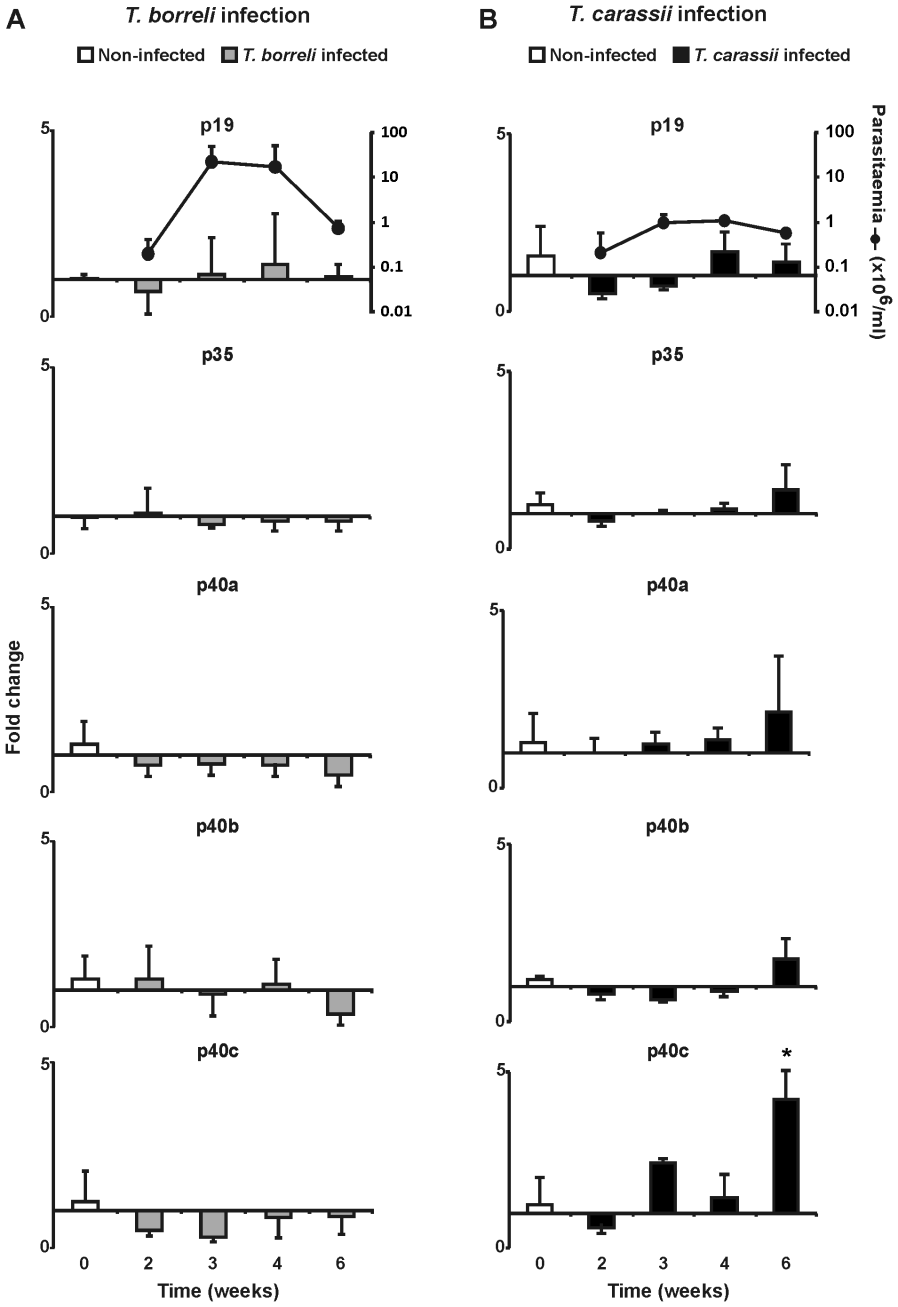
Kinetics of gene expression of IL-12 family members in head kidney during infection with *T. borreli* (A) or *T. carassii* (B). Carp were injected (i.p.) with a dose of 10000 parasites per fish, PBS-injected individuals served as negative controls (week 0). At indicated time points *n* = 5 (*T. borreli*) or *n* = 4 (*T. carassii*) animals were sacrificed for organ collection. Parasitaemia was monitored during infection and is shown in the upper plot, at a logarithmic scale. mRNA levels of IL-12 family members are shown relative to the house keeping gene 40S ribosomal protein S11. Data points represent averages + SD of *n* = 4-5 fish per time point. Symbol (*) represents a significant (*P*≤0.05) difference compared to non-infected fish.

In mammalian vertebrates the IL-12 cytokine family comprises, among others, IL-12 (composed of p35 and p40 subunits) and IL-23 (composed of p19 and p40 subunits). For carp, a single p35 and three distinct p40 genes (named p40a, p40b and p40c) have been described [6]. We cloned a partial cDNA of carp p19, comprising 255 nucleotides (GenBank accession number: HM231139) with 77% sequence similarity to the zebrafish p19 gene (GenBank accession number: ACC77208) and designed primers for gene expression studies. We subsequently examined, in head kidneys of infected fish, changes in gene expression of p19, p35 and p40a–c during *in vivo* infection with two different protozoan parasites. Infection with *T. borreli* did not induce significant changes in gene expression in none of the IL-12 cytokine family member subunits during the first two weeks of infection [8] as well as at later time points examined (Fig. 2A). In contrast, infection with *T. carassii* induced a significant 5 fold up-regulation of p40c (Fig. 2B), at least at 6 weeks post-infection.

To determine whether IL-12 cytokine family members were simply not regulated or selectively altered during protozoan infections, *ex vivo* recall responses to both parasites were performed. Head kidney leukocytes (HKL) from (non-)infected fish were stimulated *ex vivo* with PGN or PMA (positive controls), or with the homologous live parasite to assess the inducibility of the different IL-12 family members. In HKL from non-infected control fish, stimulation with PGN up-regulated p35, p40a, p40b, but not p40c nor p19. Stimulation with PMA induced a 5 fold up-regulation of p40c gene expression (data not shown). Neither PGN, nor PMA up-regulated gene expression of p19. In non-infected fish, stimulation with live parasites up-regulated p40b but not p19, p35, p40a nor p40c gene expression, independent of the protozoan parasite species. Thus, the recall responses of HKL of non-infected fish showed induced gene expression of the IL-12 family members p35 and p40, but not p19.

**Figure 3:**
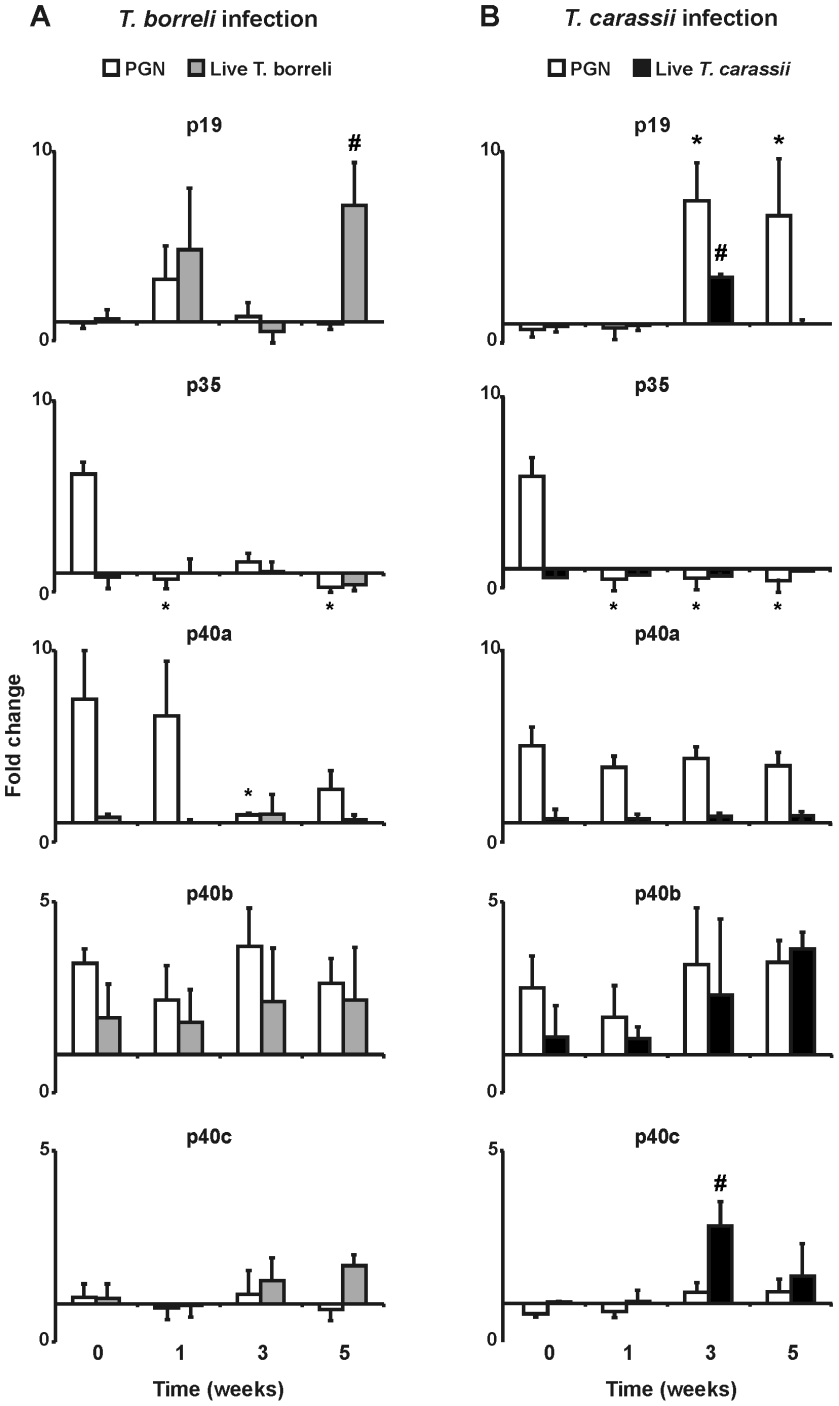
Kinetics of gene expression of IL-12 family members in re-stimulated head kidney leukocytes of *T. borreli*- (A) or *T. carassii*- (B) infected carp. Carp were injected (i.p.) with PBS or a dose of 10000 parasites per fish. Head kidney leukocytes (HKL) from non-infected (*n* = 1) or infected fish (*n* = 3) were isolated at different time points post-infection and re-stimulated with PGN (50 µg/mL) or homologous live parasites (0.25 million parasites per well) for 6 h. mRNA levels of IL-12 family members were normalized against the house keeping gene 40S ribosomal protein S11 and shown as fold change relative to unstimulated head kidney leukocytes. Data points represent averages + SD of *n* = 3 non-infected fish (taken at the three time points analysed and indicated as week 0) and *n* = 3 infected fish per time point. Symbol (*) represents a significant (*P*≤0.05) difference between PGN re-stimulated HKL from infected fish compared to PGN re-stimulated HKL from non-infected fish. Symbol (#) represents a significant (*P*≤0.05) difference between parasite re-stimulated HKL from infected fish compared to parasite re-stimulated HKL from non-infected fish.

*Ex vivo* re-stimulation of HKL from infected fish with the carp TLR2 ligand PGN [18] provides information on the degree of (non)responsiveness acquired by HKL during infection. *Ex vivo* re-stimulation with PGN did not significantly increase p19 gene expression during *T. borreli* infection. In contrast, *ex vivo* re-stimulation with PGN increased p19 gene expression in cells from fish infected with *T. carassii* at week 3 and 5 of infection (PGN, Fig. 3A and B). *Ex vivo* re-stimulation with PGN revealed a down-regulation of p35 gene expression in cells from infected fish, suggesting a suppression of expression of the p35 subunit during the whole period of *T. borreli* and *T. carassi* infections. *Ex vivo* re-stimulation with PGN increased p40a gene expression only in cells taken from fish early (1 week) during *T. borreli* infection, whereas in cells from fish infected with *T. carassii*, PGN increased p40a gene expression during the whole period of infection. *Ex vivo* re-stimulation with PGN showed that p40b gene expression was not influenced by infection with any of the two protozoan parasites, suggesting that p40b is not actively regulated during both protozoan parasite infections. *Ex vivo* re-stimulation with PGN (or with PMA) did not increase p40c gene expression in infected fish.

*Ex vivo* re-stimulation of HKL from infected fish with the homologous parasite provides information on the selectiveness of (non)responsiveness of the different IL-12 family members, acquired during infection (Fig. 3A and B). Re-stimulation of HKL with live *T. borreli* parasites significantly increased p19 gene expression (at week 5). Re-stimulation with *T. borreli* did not increase gene expression of p35 nor p40a. Gene expression of p40b or p40c was not affected by *T. borreli* infection (Live *T. borreli*, Fig. 3A). Re-stimulation of HKL with live *T. carassii* parasites significantly increased p19 (at week 3), but not p35 gene expression. Re-stimulation with live *T. carassii* parasites did not increase p40a gene expression, nor affected p40b gene expression. Re-stimulation of HKL with live *T. carassii* parasites, but not PGN, increased p40c gene expression at week 3 (Live *T. carassii*, Fig. 3B).

These results confirm the suppression of p35 gene expression during the whole period of protozoan infections and the propensity to up-regulate p19 gene expression at specific time-points of infection by both parasites. In addition, p40a gene expression was selectively impaired during *T. borreli* infection and never induced by *T. carassii* parasites. Re-stimulation of HKL with live parasites, similar to PGN, readily induced p40b gene expression, suggesting that p40b is not regulated during infection with protozoan parasites. In contrast, p40c gene expression in HKL could only be re-stimulated by live *T. carassii* parasites.

### Regulation of gene expression of different IL-12 family members by live protozoan parasites is TLR2-mediated

**Figure 4:**
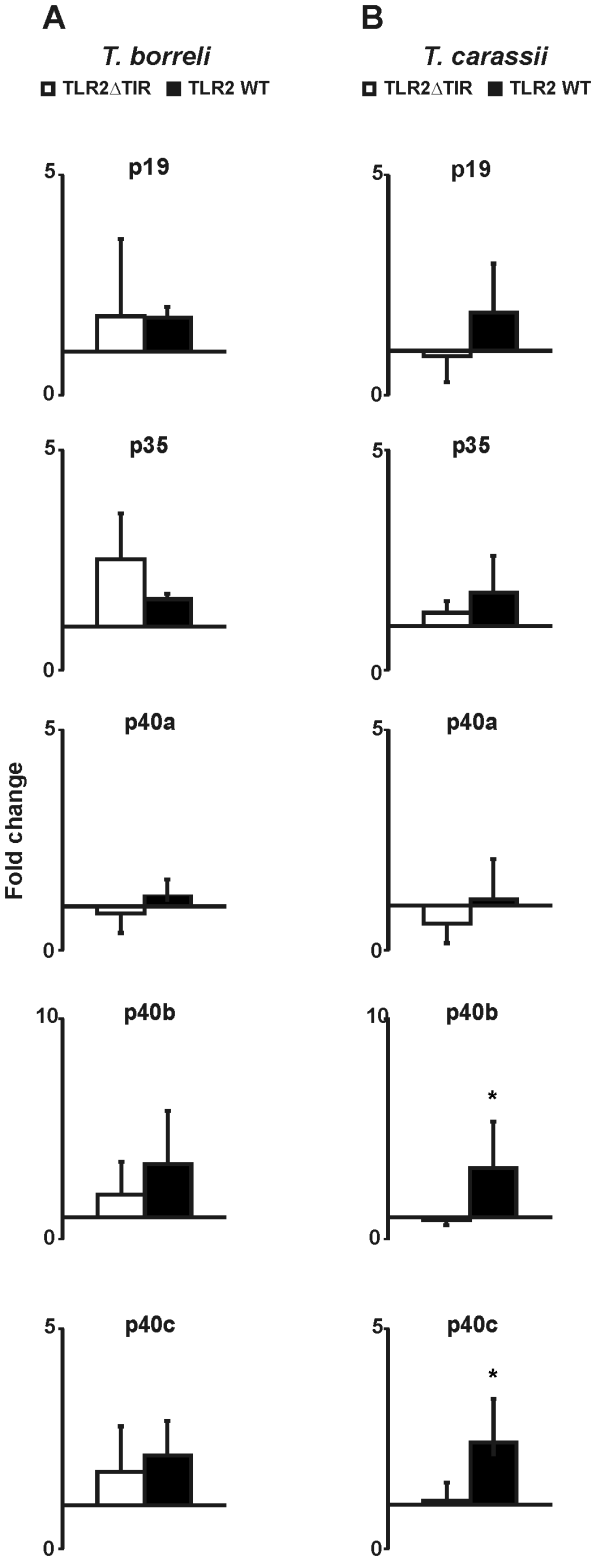
Overexpression of TLR2 in carp macrophages. TLR2ΔTIR- and TLR2WT-transfected carp macrophages were stimulated with live *T. borreli* or *T. carassii* parasites (0.25 million parasites per well) for 6 h, or left untreated as negative control. mRNA levels of IL-12 family members were normalized against the house keeping gene 40S ribosomal protein S11 and are shown as fold change relative to unstimulated macrophages (fold change  = 1). Bars show averages + SD of *n* = 4 fish. Symbol (*) represents a significant (*P*≤0.05) difference in gene expression between parasite-stimulated cells macrophages in TLR2WT-transfected- compared to TLR2ΔTIR- transfected carp macrophages.

Transfection of HEK 293 cells with TLR2WT established the ability of carp TLR2 to recognize protozoan parasite, in particular *T. carassii*-derived PAMPs (see Fig. 1). To verify the association between TLR2 activation and downstream gene transcription of the different IL-12 family members, we overexpressed TLR2 in carp macrophages. Expression of both TLR2WT and TLR2ΔTIR constructs were confirmed after transfection by western blot using anti-GFP antibody (data not shown). Stimulation of macrophages overexpressing TLR2ΔTIR was used as negative control (TLR2ΔTIR, see Fig. 4).

Stimulation of carp macrophages overexpressing TLR2WT with live *T. carassii* parasites could significantly increase p40b gene expression but not p35 nor p40a gene expression. In contrast, gene expression of the IL-12 family members p19 (*P* = 0.06) and p40c was solely induced by live *T. carassii* parasites (TLR2WT, Fig. 4A and B). Transfection of HEK293 cells with TLR2WT showed that live *T. carassii* and GPI-anchors from *T. carassii* parasites can signal through carp TLR2. Overexpression of TLR2WT in carp macrophages confirmed a TLR2-mediated p19 and p40c regulation by the protozoan parasite *T. carassii*.

### The protozoan parasite *T. carassii*, in particular, induces a Th17-like gene expression profile

Enhanced expression of the IL-12 gene family members p19 and p40c associated with *T. carassii* infections suggested this protozoan parasite, in particular, could induce an IL-23-like molecule in carp. The spleen is a secondary immune organ that increases considerably in size especially during *T. borreli* infections but also during *T. carassii* infections, indicative of an involvement in the immune response against these two parasites. To verify parasite-induced immune responses downstream of IL-23, we studied gene expression in the spleen of infected carp as a measure of a Th17-mediated immune response.

Both parasites induced gene expression of the chemokines CXCb in spleen. However, infection with *T. carassii* also significantly induced CXCL8_L2 (a chemokine related to mammalian IL-8, [28]) in spleen leukocytes. Similar to the previous experiments, gene expression of p19 and p40c was significantly different between *T. borreli* and *T. carassii*-infected fish, with p40c being significantly up-regulated only in splenocytes of *T. carassii*-infected fish. Furthermore, *T. carassii* infections were characterized by an increase in gene expression of major histocompatility class II genes and the matrix metalloproteinase MMP-9 (Table 2).

**Table 2:** Gene expression of spleen leukocytes of parasite-infected carp relative to non-infected fish.

Gene	Control^a^	*T. borreli* a	*T. carassii* a
IL-1β	1.11	1.19	2.80
TNF-α	1.22	2.02	2.28
p19#	0.84	0.84	1.61
p35	1.04	0.78	1.53
p40a	1.29	1.14	1.60
p40b	1.42	1.03	2.10
p40c#	1.05	1.17	7.02*
CXCa	1.19	1.26	1.47
CXCb	1.50	3.25*	3.08*
CXCL8_L2	1.94	4.37	4.29*
CXCR1	1.31	1.83	2.97
CXCR2	1.01	0.65	1.69
MHC-II DAB1-2	1.02	0.79	1.71*
MHC-II DAB3-4	1.01	1.16	1.80*
MMP-9	1.40	2.31	4.11*

a Averages (n = 4) of 3-weeks *T. borreli* or *T. carassii* infected fish or non-infected fish (control) are shown.

*Significant difference compared to control.

# Significant difference between *T. borreli* and *T. carassii* infected fish.

mRNA levels of the analysed genes were normalized against the house keeping gene 40S ribosomal protein S11 and are shown as fold change relative to non-infected fish.

### The protozoan parasite *T. carassii* induces neutrophilia in spleen

**Figure 5:**
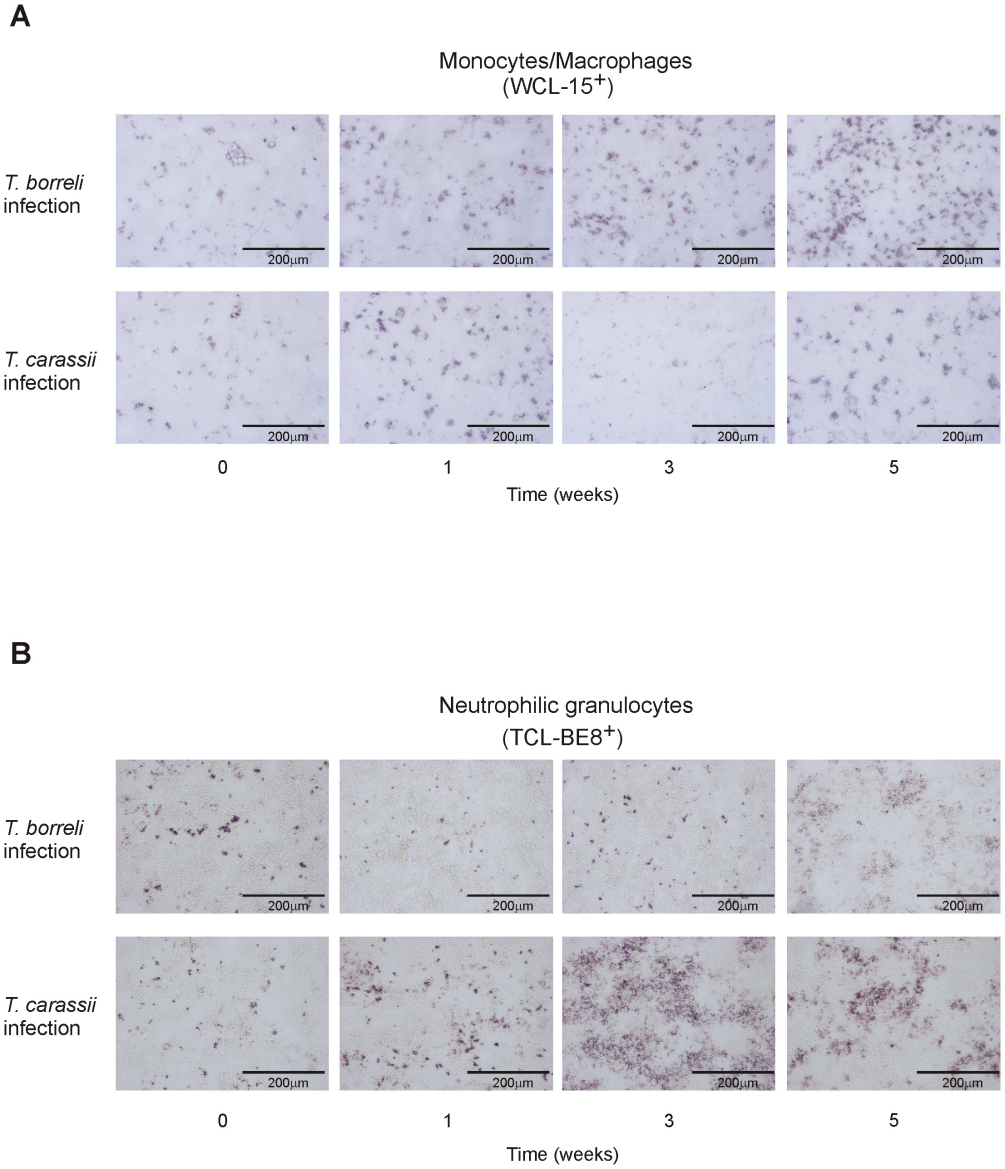
Macrophage (A) and neutrophilic granulocyte (B) cell populations in spleen of *T. borreli*- and *T. carassii*- infected fish. Carp were injected (i.p.) with a dose of 10000 parasites per fish, PBS-injected individuals served as negative controls. Spleens from non-infected and infected fish were collected at different time points post-injection. Monocytes/macrophages (WCL-15+) and neutrophilic granulocytes (TCL-BE8+) are stained in red. One experiment representative of three independent experiments is shown.

Up-regulation of gene expression of the IL-12 gene family members p19 and p40c, in combination with up-regulation of gene expression of chemokines (CXCb and CXCL8_L2) and of the matrix metalloproteinase MMP-9, suggested infections with *T. carassii* could be associated with a Th17-like immune response typically associated with neutrophilia. To investigate this hypothesis, splenic tissue from infected fish was stained for the presence of macrophages (WCL-15^+^ cells) versus the presence of neutrophilic granulocytes (TCL-BE8^+^ cells). Macrophages and neutrophilic granulocytes were present as scattered single cells in non-infected fish (Fig. 5A and 5B). During infection with *T. borreli* a clear but moderate increase in the number of macrophages was observed from week 3 (peak of parasitaemia) of infection onwards whereas a moderate increase in the number of neutrophilic granulocytes was seen at weeks 5 post-infection only. In contrast, in *T. carassii*-infected fish, a decrease in the number of macrophages was observed at week 3 post-infection, whereas a striking increase in the number of neutrophilic granulocytes was observed, often as TCL-BE8^+^ aggregates, from week 1 onwards with a peak at 3 weeks post-infection.

### Neutrophilic granulocytes from spleen of *T. carassii*-infected fish show a high p19 and p40c gene expression

**Figure 6:**
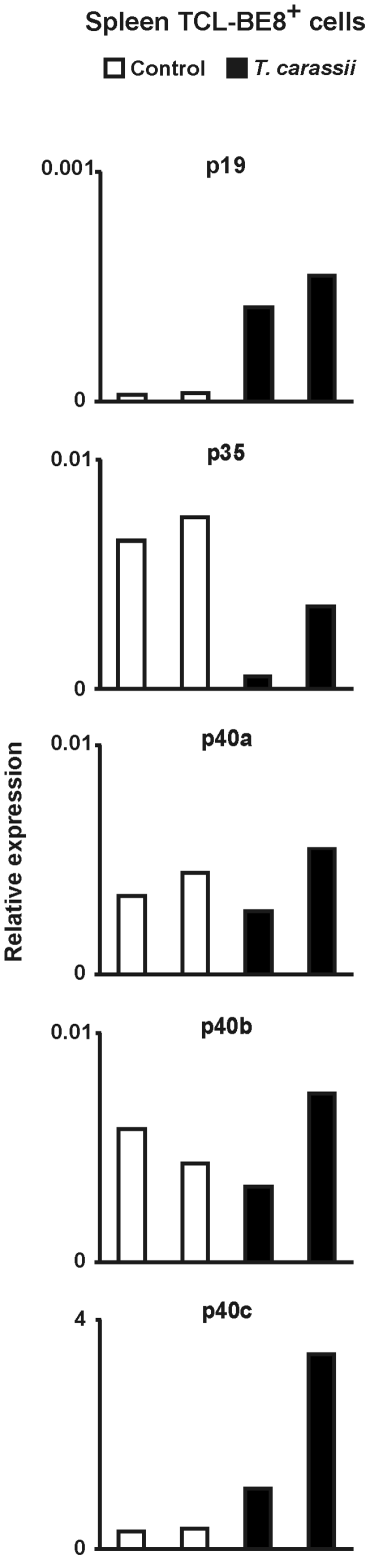
Constitutive gene expression of IL-12 family members in neutrophilic granulocytes from *T. carassii*- infected fish. Carp were injected (i.p.) with a dose of 10000 parasites per fish, PBS-injected individuals served as negative controls. Three weeks post-infection, splenocytes from non-infected (*n* = 2) or *T. carassii*-infected fish (*n* = 2) were isolated and neutrophilic granulocytes (TCL-BE8+ cells) sorted to purify by magneting sorting. Each bar represents a single fish.

The number of neutrophilic granulocytes markedly increased during *T. carassii* infection, as judged by histological examination. Splenic neutrophilic granulocytes (TCL-BE8^+^ cells) were collected from *T. carassii*-infected fish (week 3) and sorted to purity (>90%, assessed by flow cytometry) for measurement of constitutive gene expression levels of the IL-12 gene family members. Splenic neutrophilic granulocytes from *T. borreli*-infected fish were not altered at week 3 and not examined. For most of the IL-12 cytokine family genes, the level of gene expression in neutrophilic granulocytes isolated from spleen of infected fish was comparable to that seen in spleen of non-infected fish (Fig. 6). However, a high constitutive expression level of the p19 and p40c genes was observed in splenic neutrophilic granulocytes from *T. carassii*-infected fish when compared to non-infected fish.

### IFN-γ and IL-17A/F gene expression are differentially regulated during *T. borreli* and *T. carassii* infections

**Figure 7:**
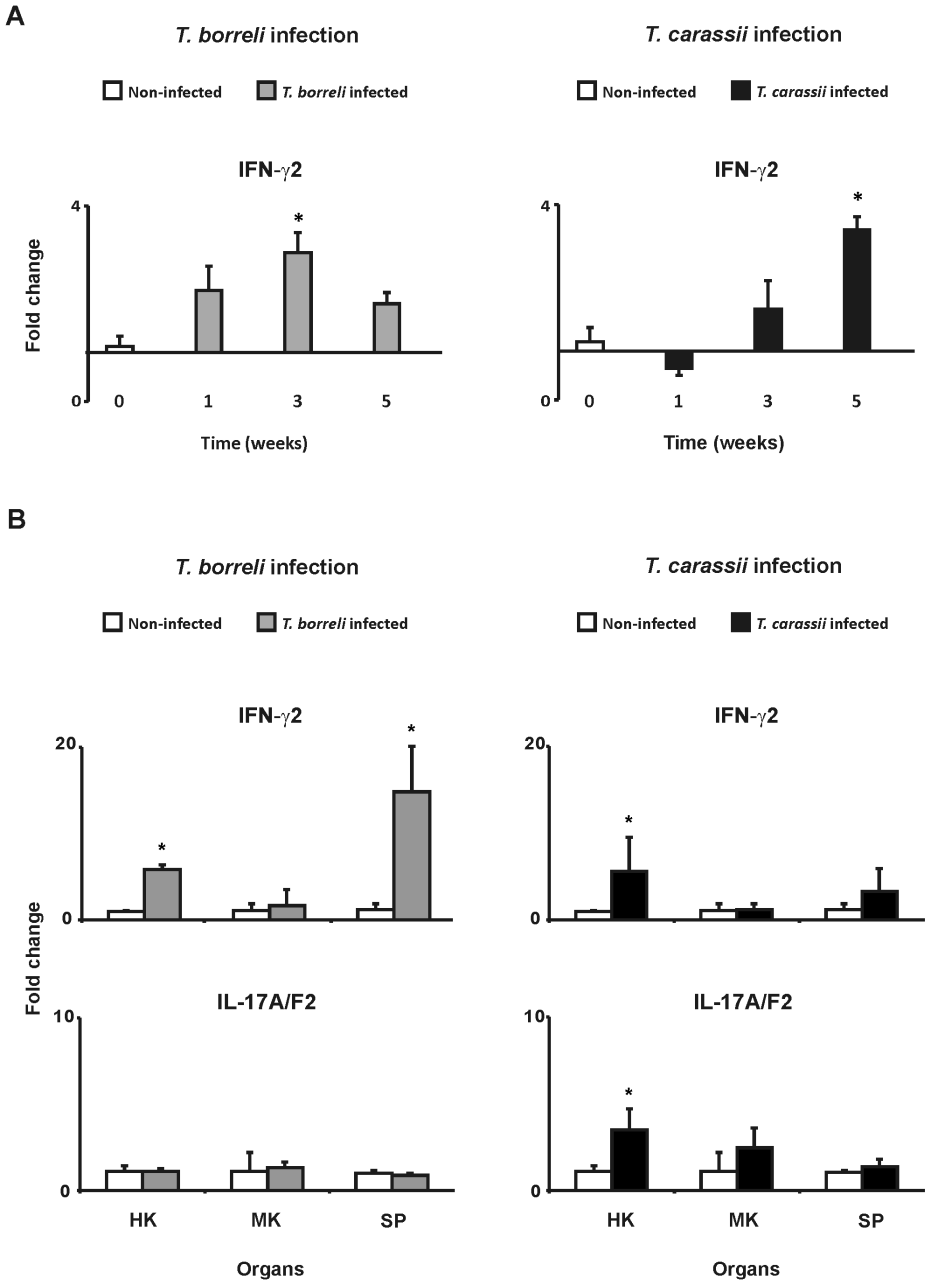
IFN-γ2 and IL-17A/F2 gene expression during *T. borreli*- and *T. carassii*-infection. Gene expression was measured at different time points post-infection in peripheral blood leukocytes (PBL) (**A**) or at a fixed time point (3 weeks) in head kidney (HK), mid kidney (MK) and spleen (SP) (**B**). Carp were injected (i.p.) with a dose of 10000 parasites per fish, PBS-injected individuals served as negative controls. mRNA levels of IFN-γ2 and IL-17A/F2 were normalized against the house keeping gene 40S ribosomal protein S11 and expressed as fold change relative to non-infected fish. Data points represent averages + SD of *n* = 3 non-infected fish (indicated as week 0) and *n* = 3 infected fish per time point. Symbol (*) represents a significant (*P*≤0.05) difference compared to non-infected fish.

In *T. carassii*-infected fish, up-regulation of gene expression for CXCb and CXCL8_L2 chemokines coincided with a marked increase of neutrophilic granulocytes in the spleen. Chemokine-dependent neutrophil recruitment, promoted by IL-17A/F, is characteristic of Th-17-mediated immune responses. For carp, two IFN-γ genes have been described, of which IFN-γ2 is associated with T cell responses [21]. We cloned a partial cDNA of carp IL-17A/F2 (GenBank accession number: HM231140), comprising 173 nucleotides with 90% sequence similarity to the zebrafish IL-17A/F2 gene and 11 and 13% sequence similarity to the zebrafish IL-17A/F1 and IL-17A/F3 genes, respectively (GenBank accession numbers:NP_001018623 [A/F1] NP_001018634 [A/F2] NP_001018626 [A/F3]) and designed primers for gene expression studies. To evaluate the development of a Th1- or Th17-mediated immune response during protozoan parasite infection of carp, we measured IFN-γ2 and IL-17A/F2 gene expression, respectively. Gene expression studies in PBL showed an upregulation of IFN-γ2 gene expression in PBL of *T. borreli*-infected fish that correlated with parasitaemia, with a peak at 3 weeks post infection. In contrast, upregulation of IFN-γ2 gene expression in PBL of *T. carassii*-infected fish was observed only at a late time point (week 5) (Fig. 7A). Transcription of IL-17A/F2 was undetectable in PBL (data not shown). Gene expression studies in head kidney and spleen showed an up-regulation of IFN-γ2 gene expression in both organs from *T. borreli*-infected fish, but only in head kidney from *T. carassii*-infected fish. Transcription of IL-17A/F2 was not regulated in immune organs of *T. borreli-*infected fish. In contrast, a significant increase of IL-17A/F2 gene expression was observed in head kidney of *T. carassii-*infected fish (Fig. 7B).

## Discussion

In our previous studies on the immune response of carp to protozoan parasites, we have measured a characteristically high production of NO by classically activated macrophages (‘type 1 response’) induced by *T. borreli*. Classically activated macrophages are antagonized by type 2 anti-inflammatory responses, which are driven by the development of alternatively activated macrophages. In our previous studies we have measured a preference for an alternative activation of macrophages (‘type 2 response’) induced by *T. carassii* [24]. Thus, these two protozoan parasites, though both extracellular blood parasites, induce different immune responses in the carp host. In the present study we have refined our investigations and conclude that infections with *T. carassii*, in particular, are associated with a Th17-like immune response. In support of this conclusion are: 1) simultaneous up-regulation of p19 and p40c gene expression in *T. carassii*-restimulated HKL and in carp TLR2-transfected macrophages stimulated with *T. carassii*, suggestive of the formation of the cytokine IL-23, 2) a strong neutrophilia in the spleen of *T. carassii*-infected fish, coinciding with the presence of chemokines and metalloproteinase MMP9, leading to a high number of neutrophils that express p19 and p40c, 3) induction of IL-17A and IL-17F, as suggested by the up-regulation of IL-17A/F2 gene expression in head kidney of *T. carassii*-infected fish.

Several studies in mammalian vertebrates have shown the involvement of TLR2 in recognition of GPI-anchors derived from protozoan parasites [29] including *Leishmania* spp [30], *T. gondii* [31], *T. cruzi* [26] and *P. falciparum* [32]. In our studies, the carp TLR2 receptor, when transfected into human cells (HEK 293) mediated the recognition of protozoan parasite-derived PAMPs. Similar to mammalian vertebrate TLR2, carp TLR2 recognizes both live protozoa as well as protozoan-derived GPI anchors, especially GPI anchors from *T. carassii*, and triggers phosphorylation of MAPK-p38 (this study). Overexpression of TLR2 in carp macrophages not only confirmed recognition of protozoan PAMPs by TLR2 but also established a TLR2-mediated p19 and p40c (IL-23) up-regulation by the protozoan parasite *T. carassii*. In mammalian vertebrates, the balance between different IL-12 cytokine family members (IL-12, IL-23 and IL-27) during the course of an immune response can be differentially regulated downstream of particular pattern recognition receptors, such as the TLRs [17]. For example, TLR2 activation by peptidoglycan from Gram-positive bacteria potently induces p19 and thus IL-23 synthesis [15]. Provided that, also in carp, p19 gene expression correlates with the production of IL-23, our data suggest there is a link between activation by protozoan PAMPs of the TLR2 receptor on, for example carp macrophages, and downstream production of the cytokine IL-23.

The cytokine IL-23 is composed of two subunits; p19 and p40. Recently, the cDNA sequence of p19 of zebrafish, a close relative of carp was described [9]. We designed primers for gene expression studies on a carp p19 cDNA sequence. Three distinct p40 genes (p40a, p40b and p40c) had already been reported for carp [6]. The identification of multiple p40 genes in carp increases the potential for heterodimeric combinations between p19, p35 and p40 subunits [33]. It is difficult to unambiguously identify the most likely candidate, if any, of the three p40 isoforms for heterodimerization with p19 to form carp IL-23. Three-dimensional modeling based on sequence information pointed at p40a and p40b, more than the p40c isoform, as likely candidates for heterodimerization with p35, to form IL-12 [6]. Although three-dimensional modeling does not provide evidence for the p40c isoform to preferentially heterodimerize with p19 to form IL-23, the simultaneous up-regulation of gene expression of p19 and p40c in *T. carassii*-re-stimulated head kidney leukocytes and in carp TLR2-transfected macrophages stimulated with *T. carassii*, suggests that heterodimerization of the p40c subunit with p19 could preferentially lead to formation of the cytokine IL-23 in carp. Of note, the constitutive gene expression of p40c is much higher than that of the other two p40 isoforms. Despite the high constitutive expression, p40c gene expression is still inducible. This suggests that high levels of p40c proteins could be produced, maybe leading to the formation of p40c homodimers that could bind to the IL-12 receptor and behave as antagonists of IL-12 (p35, p40)-mediated responses, as observed in mammals [34]; [35]. For certain, future studies, for example with plasmids composed of the different p40 subunits fused to the p19 subunit, are required to help identify the bio-active carp IL-23 protein.

The cytokine IL-12 constitutes an important factor in the differentiation and expansion of Th1 cells and plays a pivotal role in the production of IFN-γ, which in turn activates anti-microbial activity in effector cells [10]. For that reason it can be beneficial for particular microbes to specifically suppress IL-12 (p35, p40) production. The ability of protozoan parasites to suppress IL-12 formation has been documented for leishmaniasis [36], [37] in mice and trypanosomiasis in rats [38]. We found that both protozoan parasites of carp have the ability to suppress the induction of p35 and p40a gene expression gene expression during the whole period of infection, but neither parasite affected p40b gene expression (see fig. 3). This could indicate that the formation of an IL-12 molecule by heterodimerization of p35 and p40a would be the prime target for inhibition by these protozoan parasites of carp. The constitutive gene expression of p35 (and of p19) is low compared to the constitutive gene expression of p40a-c, which could mean that minimizing p35 (or p19) expression could be enough to restrict IL-12 (or IL-23)-dependent immune responses. In the case of heterodimerization of p35 and p40b, suppression of gene expression of p35 only, could be sufficient to inhibit formation of IL-12. Despite the apparent absence of IL-12 (p35) expression during both protozoan infections in carp, we observed induction of IFN-γ2 gene expression during *T. borreli* and (to a lower extent) during *T. carassii* infection. Innate immune cells such as (natural killer) NK cells, NKT cells, γδT cells or the effector Th1 or CD8+ T cells may be responsible for the observed IFN-γ2 production [39], [40].

The development of Th17 cells from naïve T cells is dependent on antigen presentation, co-stimulatory stimulation and a specific cytokine milieu (e.g.: IL-1β, IL-6, TGF-β) with IL-23 production being important for the maintenance of Th17 cells [41]. Both IL-17A and IL-17F cytokines produced by active Th17 cells appear to be especially potent activators of neutrophils, both through expansion of the lineage through regulation of G-CSF and G-CSF receptor as well as through recruitment of high numbers of neutrophils by chemokines such as CXCL1, CXCL2 and CXCL8 [42], [43]. Studies in mice revealed that ectopic expression of IL-17 stimulates a strong neutrophilic response whereas IL-17 deficiencies are associated with neutrophil defects leading to disease susceptibility [42], [44]. Thus, the Th17-mediated immune response is characterized by an IL-17A/F-driven increase of chemokines and metalloproteinases associated with recruitment and activation of neutrophils [43]. Infection of carp with the two different protozoan parasites induced different immune responses. Infection with *T. borreli* lead to an increase of IFN-γ gene expression and a moderate increase in the number of macrophages in the spleen. In contrast, infection with *T. carassii* lead to an increase of CXCb (CXCL9-11-like chemokine), CXCL8_L2 (IL-8-like chemokine) and MMP-9 gene expression and a marked increase in the number of neutrophilic granulocytes in the spleen. The concomitantly high p19 and high p40c gene expression in these neutrophils suggests a role for these cells in maintaining an IL-23-dependent immune response. Chemokine-dependent neutrophil recruitment suggests that the carp host responds to infections with *T. carassii* with a Th17-mediated immune response.

The cytokine IL-23 is required for the expansion of Th17 cells, which produce IL-17A and IL-17F but also IFN-γ [45],[13]. Three distinct IL-17A/F cDNA sequences have been identified in zebrafish [14]. We designed primers for gene expression studies on a carp IL-17 cDNA sequence. The cytokine IFN-γ exists in carp as two distinct genes with IFN-γ2, in particular, associated with characteristic T-lymphocyte function [21]. During infections with *T. borreli*, we observed an up-regulation IFN-γ2, but not of IL-17A/F2, in head kidney and spleen. In contrast, in *T. carassii*-infected fish, we observed an up-regulation of both IFN-γ2 and IL-17A/F2 in head kidney. In mammalian vertebrates, several studies indicate the presence of subsets of cells producing both IFN-γ and IL-17A/F. Double producers may represent a transitional state between Th17 and Th1 [13], [46]. Furthermore, in mice it has been shown that Th17 are phenotypically unstable and readily convert to a Th1 phenotype, but not vice versa [47], [48]. When transferred to Th1-polarizing conditions and in the absence of IL-23, Th17 cells decrease synthesis of the IL-23R chain, thus allowing the reconstitution of the heterodimeric IL-12 receptor and promoting the production of IFN-γ. It has been suggested that T cells either co-produce IFN-γ and IL-17A/F or suppress IL-17A/F and continue to produce IFN-γ [13]. The co-induction of IFN-γ2 and IL-17A/F2 during infection with *T. carassii* suggests the presence of mixed Th1/Th17 phenotypes in *T. carassii*-infected fish. Although host defense against extracellular bacteria is widely considered to be dominated by a Th17 response [49], a mixed Th1/Th17 polarization was observed during *Bordetella pertussis* infection [50]. The fact that IFN-γ2 is induced especially at later stages of the *T. carassii* infection suggests that carp IFN-γ could play a role in limiting Th17-mediated responses [51]. Therefore, efficient protection against the extracellular protozoan parasite *T. carassii* in carp may require synergy between Th17- and Th1-mediated cell responses.

In mammalian vertebrates, IL-12 is important for the differentiation and expansion of Th1 cells and production of IFN-γ against intracellular bacteria and parasites. IL-23 is important for the expansion of Th17 cells, which produce IL-17 in response to extracellular bacteria and fungi. There is some evidence regarding the role of Th17-mediated responses to protozoan parasites, but it is limited. Infection of IL-17-deficient mice with *Toxoplasma gondii* lead to a higher mortality than in control mice [52]. In contrast IL-27, which suppresses Th17 responses, was beneficial for the host during *Trypanosoma cruzi* infection [53]. There are several studies that implicate TLR2 activation in p19 gene expression [54], neutrophil transmigration [55] and promotion of Th17 responses [56]. Our studies show that *T. carassii*-derived PAMPs are agonists of carp TLR2, promoting p19 and p40c gene expression. This is the first study that provides evidence for a Th17-like immune response in fish to infection with protozoan parasites.

## Materials and Methods

### Ethic statement

All animals were handled in strict accordance with good animal practice as defined by the relevant national and/or local animal welfare bodies, and all animal work was approved by the animal experimental committee of Wageningen University, Wageningen, The Netherlands. (license numbers: 2004079/2004137/2008054).

### Animals

European common carp (*Cyprinus carpio carpio* L.) were reared in the central fish facility of Wageningen University, The Netherlands at 23°C in recirculating UV-treated tap water and fed pelleted dry food (Skretting, Nutreco) daily. R3xR8 carp are the hybrid offspring of a cross between fish of Hungarian origin (R8 strain) and of Polish origin (R3 strain) [57]. Carp were between 9 and 11 months old at the start of the experiments.

### Protozoan parasites and GPI-anchors

*Trypanoplasma borreli* was cloned and characterized by Steinhagen *et al*. [19]. *Trypanosoma carassii* was cloned and characterized by Overath *et al.* [58]. Both parasites were maintained by syringe passage through carp. Parasitaemia was monitored in 10 x diluted blood in cRPMI [RPMI 1640 (Invitrogen, CA, USA) adjusted to carp osmolarity 280 mOsmkg^−1^ containing 50 U/ml of heparin (Leo Pharma BV, Weesp, The Netherlands)] using a Bürker counting chamber. The minimum detection limit by this method was 10^5^ parasites/ml of blood. For parasite isolation, blood was collected from 3-weeks-infected fish and purified on a 1×12cm ion-exchange chromatography using DEAE cellulose (DE-52; Whatman international) [58]. After purification, parasites were resuspended in HML medium [59] supplemented with 5% pooled carp serum, L-glutamine (2 mM, Cambrex, Verviers, Belgium), penicillin G (100 U/ml, Sigma-Aldrich, Zwijndrecht, The Netherlands), and streptomycin sulfate (50 mg/l, Sigma-Aldrich).

GPI-anchors from the parasites were obtained as described previously [18]. In short, equal numbers of *T. borreli* or *T. carassii* parasites were incubated for 30 min at 30°C in 30% Tris-buffer (10 mM Tris-HCl, 144 mM NaCl, 0.05% BSA, pH = 7.4) or in 1 unit of phosphatidylinositol-specific phospholipase C [PI-PLC from *Bacillus cereus* (Sigma-Aldrich)] in 30% Tris-buffer. Samples were centrifuged at 800 *g* for 10 min and supernatants were collected and filter-sterilized (0.22 µm Millex-GV, Millipore, Ireland). Pellets from PI-PLC-treated parasites were collected and resuspended in incomplete NMGFL-15 medium. Carp macrophages were stimulated with 25 µL (1∶4) of each fraction. HEK 293 cells were stimulated with 50 µL (1∶5) of each fraction.

### Experimental setup of infection of carp with protozoan parasites

*In vivo* infections with *T. borreli* or *T. carassii* were performed as described previously [8] and [24], respectively). Before the start of each infection experiment fish were moved to a quarantine facility and acclimatized to 20°C over a period of at least 2 weeks. Fish were euthanized with 0.3 g/l tricaine methane sulfonate (TMS, Crescent Research Chemicals, Phoenix, AZ, USA) buffered with 0.6 g/l NaHCO_3_ (Merck, Darmstadt, F.R. Germany) prior to sampling blood, head kidney or spleen.

For gene expression studies in parasite-infected fish, carp were i.p. injected with 10^4^ *T. borreli* parasites per fish in 100 µL or with PBS (unchallenged control group) and head kidneys of n = 5 infected and n = 3 non-infected fish sampled per time point, over a period of 6 weeks post-infection. In a separate experiment, carp were i.p. injected with 10^4^ *T. carassii* parasites per fish in 100 µL or with PBS and head kidneys of n = 4 (non-) infected fish sampled per time point, over a period of 10 weeks.

### Recall experiments with parasite-infected carp

For *ex vivo* recall experiments with phagocytes from parasite-infected carp, carp were i.p. injected with 10^4^ parasites (*T. borreli* or *T. carassii*) per fish in 100 µL, or with PBS, and head kidneys of n = 3 infected and n = 1 non-infected fish sampled per time point, over a period of 5 weeks. Blood was collected by puncture of the caudal blood vessel by a syringe containing heparinised RPMI 1640; a small aliquot of blood was used to determine parasitaemia using a Bürker counting chamber and the remainder was used to isolate peripheral blood leukocytes (PBL). At the same time, spleens and head kidneys were aseptically removed. Spleens were snap frozen in liquid nitrogen and stored at −80°C until use. Head kidneys were used to isolate head kidney leukocytes (HKL).

For PBL isolation, blood was centrifuged first for 15 min at 800 *g* to remove the red blood cells. The buffy coat containing the leukocytes was collected and layered on 3 ml Ficoll-Paque™ Plus (Amersham Biosciences, Uppsala, Sweden). Following subsequent centrifugation at 800 *g* for 25 min with the brake disengaged, leukocyte layer at the interface was collected and washed three times with cRPMI. Cell pellets were collected, directly lysed and stored at −80°C prior to RNA isolation.

HKL isolation was performed as previously described [25]. Briefly, head kidneys were gently passed through a 100 µm sterile nylon mesh (BD Biosciences, Breda, The Netherlands) and rinsed with homogenization buffer [incomplete NMGFL-15 medium containing 50 U/ml penicillin G, 50 µg/ml streptomycin sulphate, and 20 U/ml heparin. Cell suspensions were layered on 51% (1.07 g.cm^−3^) Percoll (Amersham Biosciences, Uppsala, Sweden) and centrifuged at 450 *g* for 25 min at 4°C with the brake disengaged. HKL at the interphase were collected and washed twice in incomplete NMGFL-15. *Ex vivo* recall stimulations were performed with HKL (0.5×10^6^ per well) seeded in 100 µL rich-NMGFL-15 medium [incomplete NMGFL-15 medium supplemented with 2.5% heat-inactivated pooled carp serum and 5% fetal bovine serum (FBS, Invitrogen)] in a 96-well culture plate. HKL were stimulated with peptidoglycan (50 µg/mL, soluble secreted PGN from *S. aureus*, Invivogen, Cayla SAS, France), live *T. borreli* or *T. carassii* (0.25×10^6^ parasites per well) for 6 h at 27°C prior to RNA isolation, or left untreated.

### Macrophage cell cultures

Head kidney-derived macrophages, considered the fish equivalent of bone marrow-derived macrophages, were prepared as previously described [25], [60]. Briefly, carp head kidneys were gently passed through a 100 µm sterile nylon mesh, rinsed with homogenization buffer. Cell suspensions were layered on 51% (1.07 g.cm^−3^) Percoll and centrifuged at 450 *g* for 25 min at 4°C with the brake disengaged. HKL at the interphase were removed and washed twice in incomplete NMGFL-15 medium. Macrophage cell cultures were initiated by seeding 1.75×10^7^ head kidney leukocytes in a 75 cm^2^ culture flask containing 20 ml of complete NMGFL-15 medium [incomplete NMGFL-15 medium supplemented with 5% heat-inactivated pooled carp serum and 10% FBS]. Head kidney-derived macrophages, named macrophages throughout the manuscript, were harvested after 6 days of incubation at 27°C by placing the flasks on ice for 10 min prior to gentle scraping.

### Purification of neutrophilic granulocytes

Total leukocytes from spleen were isolated essentially as described for head kidney total leukocytes [61]. Cell suspensions were layered on a discontinous Percoll gradient (1.020 and 1.083 g cm^−3^) and centrifuged 30 min at 800 *g* with the brake disengaged. Cells at the interface 1.020 and 1.083 g.cm^−3^ were collected and washed twice with cRPMI. The monoclonal antibody TCL-BE8 (1∶50) [62] was used to purify neutrophilic granulocytes by magnetic sorting [18]. After incubation for 30 min with TCL-BE8 on ice, the leukocyte suspension was washed twice with cRPMI and incubated with phycoerythrin (PE)-conjugated goat anti-mouse (1∶50; DAKO, Glostrup, Denmark) 30 min on ice. After washing twice, the total cell number was determined with a Bürker counting chamber, and 10 µl of magnetic beads (anti-PE Microbeads, Miltenyi Biotec, GmbH, Germany) was added per 10^8^ cells. After incubation for 15 min at 4°C, cells were washed twice and resuspended in cRPMI. The magnetic separation was performed on LS-MidiMACS Columns (Mitenyi Biotec) according to the manufacturer's instructions. For RNA isolation, TCL-BE8^+^ cells were resuspended in 1 ml of cRPMI and directly lysed. The purity of the TCL-BE8^+^ neutrophilic granulocyte-enriched fraction was >90% as confirmed by flow cytometric analysis using a FACScan® flow cytometer (Becton Dickinson, Mountain View, CA, USA).

### Immunohistochemistry

Cryosections (7 µM) of spleen tissue were mounted on poly-*L*-lysine-coated glass slides (BDH Laboratory Supplies, Poole, UK), air-dried for 60 min and incubated in a 0.3% H_2_O_2_ solution in methanol for 20 min to inactivate endogenous peroxidase. Following steps were performed at RT unless stated otherwise. Cryosections were washed for 5 min with PBS, then short with distilled water and incubated in proteinase-K solution (50 µg/ml in distilled water) for 10 min at 37°C. Samples were fixed in 4% paraformaldehyde in PBS for 10 min at 4°C followed by washing in 0.1% Triton PBS (PBS-T) for 10 min at 4°C and subsequently in PBS-T for 7 min at RT. Cryosections were first blocked in 5% normal goat serum for 30 min and then incubated with primary antibody in PBS for 1 h. Mouse monoclonal antibodies were used to detect neutrophilic granulocytes (TCL-BE8, 1∶50) [62] and macrophages (WCL-15, 1∶50) [63], [64]. After washing twice for 10 min in PBS-T, sections were incubated with goat anti-mouse horseradish peroxidase-conjugated (GAM-HRP, 1∶200, Dako, Glostrup, Denmark) in PBS for 1 h. After washing twice in PBS-T, sections were incubated for 10 min in 0.05 M sodium acetate buffer, pH 5 and following addition of 0.4 mg/ml 3-amino-9-ethyl-carbazole (AEC; Sigma-Aldrich) in sodium acetate buffer containing 0.03% H_2_O_2_ and incubated for 25 min. Finally, cryosections were rinsed four times in distilled water and embedded in Kaiser's glycerine gelatin (Merck, Darmstadt, Germany).

### TLR2 WT-GFP and TLR2ΔTIR-GFP expression plasmids

Amplification of carp TLR2WT (wild type; full-length sequence) and TLR2ΔTIR (sequence truncated at TIR domain) cDNA was performed as previously described [18]. The vivid color™pcDNA™6.2/C-EmGFP-GW/TOPO® (Invitrogen, catalog no. K359-20) expression vector combined with TOPO®cloning was used to fluorescently label the construct by fusing TLR2 WT or TLR2ΔTIR to EmGFP at the C-terminal end. Isolation of highly pure plasmid DNA suitable for transfection was performed using S.N.A.P.™ Midi Prep Kit (Invitrogen, catalog no. K1910-01), according to the manufacturer's protocol. Fluorescence-tagged protein was visualized using confocal microscopy.

### Transient transfection of HEK 293 cells and carp macrophages

HEK 293 cells were cultured in DMEM supplemented with 10% FBS, 50 U/ml penicillin G and 50 µg/ml streptomycin sulphate. Two days prior to transfection, HEK 293 cells were seeded into tissue culture flasks to reach 80-90% confluence at the day of transfection. For transfection of HEK 293 cells, 2.5 µg of the carp TLR2 WT-GFP or TLR2ΔTIR-GFP constructs was transfected into HEK 293 by nucleoporation using nucleofactor™ solution V and program A-23 (Lonza Cologne AG, Germany) according to the manufacturer's instructions. Forty-eight hours after transfection, cells were trypsinized (0.5% trypsin, GIBCO) and plated overnight in a 24-well plate [18]. The next day, cells were stimulated for 15 min with live protozoan parasites *(T. borreli* or *T. carassii*; 0.5×10^6^ per well) or GPI-enriched fractions from 5×10^6^ parasites *(T. borreli* or *T. carassii*, 50 µl per well), or left untreated as negative control. Cells were lysed for evaluation of phospho-p38 activity by Western blot.

For transfection of carp macrophages, 2.5 µg of the carp TLR2 WT-GFP or TLR2ΔTIR-GFP constructs was transfected by nucleoporation using nucleofactor™ Human Macrophage Solution and program Y-001 (Lonza Cologne AG, Germany) according to the manufacturer's instructions and placed into a 48-well plate [18]. After 24 h incubation, the medium was replaced and macrophages were stimulated for 6 h with live parasites (*T. borreli* or *T. carassii*; 0.5×10^6^ per well) or GPI-enriched fractions from 5×10^6^ parasites (*T. borreli* or *T. carassii*, 50 µl per well). Cells were lysed for RNA isolation.

### RNA isolation and cDNA synthesis

RNA was isolated using the RNeasy Mini Kit (Qiagen, Leusden, The Netherlands) including the accompanying DNase I treatment on the columns, according to the manufacturers' protocol. Final elution was performed with 30 µl nuclease-free water. RNA concentrations were measured by spectrophotometry (Nanodrop, Thermo scientific, Breda, The Netherlands) and 1 µl was analysed on a 1% agarose gel to check the RNA integrity. RNA was stored at −80°C until further use. Prior to cDNA synthesis, a second DN*ase* treatment was performed using DN*ase* I, Amplification Grade (Invitrogen). Briefly, 1 µg of RNA from each sample was combined with 10X DN*ase* reaction buffer and 1 U DN*ase* I, mixed and incubated at RT for 15 min, followed by inactivation of DN*ase* I by adding 1 µl of 25 µM EDTA. Synthesis of cDNA was performed with Invitrogen's SuperScript™ III First Strand Synthesis Systems for RT-PCR Systems, according to the manufacturer's instructions. Briefly, DN*ase* I-treated RNA samples were mixed with 5x first strand buffer, 300 ng random primers, 10 µM dNTPs, 0.1 M DTT, 10 U RN*ase* inhibitor, and 200 U SuperScript III Reverse Transcriptase up to a final volume of 20 µl. The mixture was incubated at 37°C for 60 min followed by an inactivation step at 70°C for 15 min. A non-reverse transcriptase control was included for each sample. cDNA samples were further diluted 50 times in nuclease-free water before use as template in real-time PCR experiments.

### Identification of carp p19 and IL-17A/F2 cDNA

Based on previously described sequences for zebrafish p19 (GenBank Accession number: ACC77208) and zebrafish IL-17A/F1-3 (GenBank Accession numbers:NP_001018623 [A/F1] NP_001018634 [A/F2] NP_001018626 [A/F3]), primers were designed to amplify the corresponding cDNA regions for p19 and IL-17A/F2 in common carp.

cDNA from head kidney and mid-kidney from *T. carassii* infected fish was used as template for PCR to clone carp p19 and IL-17A/F2, respectively. A first PCR round was performed using the following primers to amplify p19: p19Fw-GCCTTCAAAGCAACAAAAAGACTT and p19Rv-GGAGTAGAGTCTTTCCACGCTGT. A first PCR round was performed using the following primers to amplify IL-17A/F2: IL-17A/F2: IL-17A/F2Fw-GTCTGCGTGGAACTGGATACCGAA and IL-17A/F2Rv-CAGCACCAGTATGTCCTGATAAATG. A second round using the same primer combination was performed to obtain a partial carp p19 (GenBank accession number: HM231139, 255 bp) and carp IL-17A/F2 cDNA (GenBank accession number: HM231140, 262 bp).

PCR reactions were performed in *Taq* buffer, using 1U *Taq* polymerase (Promega, Leiden, The Netherlands) supplemented with MgCl_2_ (1.5 mM), dNTPs (200 µM) and primers (400 nM each) in a total volume of 50 µl. PCR and nested PCR were carried out under the following conditions: one cycle 4 min at 96°C; followed by 35 cycles of 30 sec at 96°C, 30 sec at 55°C and 2 min at 72°C; and final extension for 7 min at 72°C, using a GeneAmp PCR system 9700 (PE Applied Biosystems, Foster City, CA). Products amplified by PCR, nested PCR or RACE-PCR were ligated and cloned in JM-109 cells using the pGEM-Teasy kit (Promega) according to the manufacturer's protocol. From each product both strands of eight clones were sequenced, using the ABI prismBigDye Terminator Cycle Sequencing Ready Reaction kit and analysed using 3730 DNA analyser.

### Real-time quantitative PCR (RT-qPCR)

Real time quantitative PCR (RT-qPCR) was performed in a 72-well Rotor-Gene™ 2000 (Corbett Research, Mortlake, Sydney, Australia) with the Brilliant® SYBR® Green QPCR (Stratagene, La Jolla, CA, USA) as detection chemistry. Primers used for RT-qPCR (Table 3) were designed with Primer Express software. IL1-β and TNF-α primer sets were designed to amplify all known isoforms for each gene. Master-mix for each PCR run was prepared as follows: 0.32 µl of water, 0.84 µl of each primer (5 µM), 7 µl Master SYBR Green I mix. To 5 µL of diluted cDNA, 9 µl of master mixed was added in a 0.1 ml tube. Following amplification program was used: one denaturation step of 15 min at 95°C; followed by 40 cycles of RT-qPCR with three-step amplification (15 s at 95°C for denaturation, 30 s at 60°C for annealing and 30 s at 72°C for elongation) and a final holding step of 1 min at 60°C. A melting step was then performed with continuous fluorescence acquisition starting at 60°C with a rate of 1°C/5 s up to 99°C to determine the amplification specificity. In all cases, the amplifications were specific and no amplification was observed in negative controls (non-template control and non-reverse transcriptase control). Fluorescence data from RT-qPCR experiments were analysed using Rotor-Gene version 6.0.21 software and exported to Microsoft Excel. The cycle threshold *C*_t_ for each sample and the reaction efficiencies (*E*) for each primer set were obtained upon Comparative Quantitation Analysis from the Rotor-Gene version 6.0.21 software. Briefly, the *E* for each primer set was recorded per sample and an average *E* (*E*_A_) was then calculated for each primer set. The relative expression ratio (*R*) of a target gene was calculated based on the *E*_A_ and the *C*_t_ deviation of sample versus control, and expressed in comparison to a reference gene [65], [66].

**Table 3:** Primers used for real-time quantitative PCR analysis.

Primer	Sequence (5′-3′)	GenBank Accession No.
40S Fw	CCGTGGGTGACATCGTTACA	AB012087
40S Rv	TCAGGACATTGAACCTCACTGTCT	
TLR2 Fw	TCAACA+CTCTTAATGTGAGCCA a	FJ858800
TLR 2 Rv	TGTG+CTGGAAA+GGTTCAGAAA a	
IL-1β Fw	AAGGAGGCCAGTGGCTCTGT	AJ245635
IL-1β Rv	CCTGAAGAAGAGGAGGAGGCTGTCA	
TNF-α1,2 Fw	GCTGTCTGCTTCACGCTCAA	AJ311800-01
TNF-α1,2 Rv	CCTTGGAAGTGACATTTGCTTTT	
p19 Fw	CTCGCTCTGAAAAACTA+CACCAGG a	HM231139
p19 Rv	GGCACGCTCTCTC+CACTTACT a	
p35 Fw	TGCTTCTCTGTCTCTGTGATGGA	AJ580354
p35 Rv	CACAGCTGCAGTCGTTCTTGA	
p40a Fw	GAGCGCATCAACCTGACCAT	AJ621425
p40a Rv	AGGATCGTGGATATGTGACCTCTAC	
p40b Fw	TCTTGCACCGCAAGAAACTATG	AJ628699
p40b Rv	TGCAGTTGATGAGACTAGAGTTTCG	
p40c Fw	TGGTTGATAAGGTTCACCCTTCTC	AJ628700
p40c Rv	TATCTGTTCTACAGGTCAGGGTAACG	
CxCa Fw	CTGGGATTCCTGACCATTGGT	AJ421443
CxCa Rv	GTTGGCTCTCTGTTTCAATGCA	
CxCb Fw	GGGCAGGTGTTTTTGTGTTGA	AB082985
CxCb Rv	AAGAGCGACTTGCGGGTATG	
CXCL8_L2 Fw	TCACTTCACTGGTGTTGCTC	AB470924
CXCL8_L2 Rv	GGAATTGCTGGCTCTGAATG	
CxCR1 Fw	GCAAATTGGTTAGCCTGGTGA	AB010468
CxCR1 Rv	AGGCGACTCCACTGCACAA	
CxCR2 Fw	TATGTGCAAACTGATTTCAGGCTTAC	AB010713
CxCR2 Rv	GCACACACTATACCAACCAGATGG	
MHC-II DAB1-2 Fw	ACAGCTCCCGTGATTTCAGT	Z47731-32
MHC-II DAB1-2 Rv	CTCTGCGTTATATATACTCCAAGTGC	
MHC-II DAB3-4 Fw	GCGTTTCAGGCGGACTCTT	Z47733
MHC-II DAB3-4 Rv	ACACCATATCACTGTAATCACT	
MMP9 Fw	ATGGGAAAGATGGACTGCTG	AB057407
MMP9 Rv	TCAAACAGGAAGGGGAAGTG	
IFNγ2 Fw	TCTTGAGGAACCTGAGCAGAA	AM168523
IFNγ2 Rv	TGTGCAAGTCTTTCCTTTGTAG	
IL-17A/F2 Fw	ATGTCCTGATAAATGGG+CAGTGAG a	HM231140
IL-17A/F2 Rv	TGTCCTGATAAATGGGCAGT+GAGT a	

a The ‘+’is before the nucleic acid in which the locked nucleic acid bond was placed.

### Western blot analysis

TLR2-transfected HEK 293 cells were used to investigate protozoan parasite ligands using phosphorylation detected with an antibody specific for phospho-p38 as a measure for responsiveness. To this purpose, HEK 293 cells were resuspended by pippeting and transferred to pre-cooled eppendorf tubes. Cells were washed twice in ice-cold PBS, lysed on ice with lysis solution [0.5% Triton X-100, 20 mM Tris, 100 mM NaCl, 1 mM EDTA, 50 mM NaF (Sigma), 1 mM phenylmethylsulfonyl fluoride (PMSF, Sigma)], homogenized with a syringe and incubated 10 min on ice. Cell lysates were centrifuged at 21000 *g* for 10 min at 4°C. Supernatant was collected and total protein content was determined by the Bradford method. Samples (20–25 µg) were boiled at 96°C for 10 min with loading buffer containing β-mercaptoethanol and separated by 10% SDS-PAGE and electrophoretically transferred to nitrocellulose membranes (Protrans, Schleicher & Schuell, Bioscience GmbH). Membranes were blocked in 3% BSA in TBS (10 mM Tris, 150 mM NaCl, pH 7.5) for 1 h at room temperature and then incubated with primary antibody overnight at 4°C in 3% BSA in TBS. Following antibodies reactive to both humans and carp were used: rabbit IgG anti-phospho-p38 (1∶1000, Thr180/Tyr182, BioCat GmBh, Heidelberg, Germany) and rabbit IgG anti-β-tubulin (1∶500, Abcam, Cambridge, UK). Membranes were then incubated with goat-anti-rabbit HRP-conjugated (1∶1000, Dako) in 10% milk powder in TBS for 1 h at room temperature. Between each incubation step, membranes were washed twice with TBS-Tween/Triton (TBS, 0.05% (v/v) Tween 20, 0.2% (v/v) Triton X-100) and once with TBS, for 10 min at RT. Signal was detected by development with a chemoluminescence kit (Amersham) according to the manufacturer's protocol and visualized by the use of Lumni-fil chemiluminescent Detection Film (Roche, Woerden, The Netherlands). The blots were scanned and saved as a greyscale TIFimages. Each image was converted to a binary image and the number of pixels in each band were quantified using a user-defined threshold in a custom-written MATLAB (release 2009a) script.

### Statistical Analysis

Transformed values (ln) were used for statistical analysis in SPSS software (version 17.0). Homogeneity of variance was analyzed using the Levene's test. Significant differences (*P*≤0.05) for the *in vivo* gene expression studies were determined by a two-way ANOVA followed by a Sidak's test. Significant differences between treatments (*P*≤0.05) for the *ex vivo* and *in vitro* gene expression studies were determined by one-way ANOVA followed by Sidak's test. In case of unequal variances between treatments, the one-way ANOVA was followed by a Games–Howell test.
